# From knowledge accumulation to transformative action: research to catalyze progress on social determinants of health equity

**DOI:** 10.1186/s12939-026-02900-4

**Published:** 2026-08-12

**Authors:** Julia Berenson, Robert Marten, Wenhui Mao, Debashis Basu, Monika Kosinska, Andrew Cassels, Gavin Yamey, Kumanan Rasanathan

**Affiliations:** 1 Department of Social Determinants of Health, WHO, Geneva, Switzerland; 2 Alliance for Health Policy and Systems Research, WHO, Avenue Appia 20, Geneva, CH-1217 Switzerland; 3 Duke University, Durham, USA; 4 University of Pretoria, Pretoria, South Africa; 5 University of Western Australia, Perth, Australia

**Keywords:** Health equity, Social determinants of health, Health policy, Health systems, Research agenda

## Abstract

Despite three decades of expanding scholarship on social determinants of health (SDH), progress in reducing health inequities has remained limited. While the SDH framework has reshaped global health discourse and informed policy rhetoric, the translation of knowledge into transformative action has been insufficient. The gap between evidence and impact is not explained by political constraints alone but is also contributed to by the orientation, methods, and practices of SDH research itself. We identify key limitations in what is studied, how research is conducted, and who leads knowledge production. Thematic gaps include the dominance of descriptive research, limited evidence on effective policy interventions, insufficient engagement with structural determinants and political economy, and underdeveloped attention to emerging drivers such as climate change, digitalization, and commercial determinants. Methodological limitations include underuse of policy, implementation, modelling, and participatory approaches, alongside persistent data gaps. Deficiencies in research practice constrain the transformative potential of SDH scholarship, including a lack of multi-disciplinary work, limited community and policymaker engagement and leadership, and inequitable power dynamics in global health research itself. We propose a reorientation of SDH research toward agency and action with greater focus on solutions and implementation pathways, methodological pluralism, multisectoral and participatory knowledge production, improved communication and policy engagement, and reforms to research incentives, funding and governance. By repositioning SDH research as an active contributor to political and institutional change, researchers can more effectively support equitable and sustainable health outcomes in a global context increasingly inhospitable for attention to health equity.

## Key messages

Despite extensive evidence on social determinants of health, research has not translated into sufficient policy action or sufficient measurable reductions in health inequities.

Dominant research approaches remain overly descriptive, insufficiently focused on solutions, and inadequately engaged with structural, political, and economic drivers of inequity.

Underuse of policy, implementation, modelling, and participatory methods, alongside limited multisectoral collaboration, inadequate engagement with political actors and cycles, and inequitable power dynamics in research, constrain impact.

SDH research must shift from documenting inequities and presenting normative recommendations to generating actionable, context-sensitive evidence that informs feasible policy design, implementation, and political strategy.

Advancing health equity requires methodological pluralism, multisectoral and community-led knowledge production, stronger engagement with policymakers, and reforms to research incentives and communication practices.

## Introduction

Over the past three decades, the social determinants of health (SDH) have become an established and influential framework for understanding health inequities [1]. Following the seminal work of the 2008 WHO Commission on Social Determinants of Health (CSDH) and a rapidly expanding body of scholarship - largely rooted in social epidemiology - there is now broad consensus that people’s daily living conditions and their differential access to power, money, and resources are responsible for most health inequities within and between populations [2]. This framing has reshaped global health discourse, entered policy language, and informed numerous strategies and declarations [3].

Despite this robust and growing evidence base on the contribution of SDH to health inequities, progress in reducing health inequities is disappointing. While some inequities between countries have narrowed - largely driven by economic growth and poverty reduction, educational and nutritional advancement, and health service improvement - inequities within many countries have widened [4–7]. This divergence is particularly striking given the exponential growth of research on SDH ^8^. In other words, knowledge has increased, but equity has not improved sufficiently (for example, the targets set by the CSDH have not been achieved) or even worsened.

Moreover, the translation of SDH discourse into concrete implementation of health, social, and economic policies is limited. Although social determinants are increasingly acknowledged in the introductions of health policy documents and rhetorical commitments, they rarely constitute the central focus of intervention efforts or receive commensurate levels of funding [8]. The health sector continues to prioritize healthcare delivery, disease-specific interventions, and individual-level risk factors. Structural drivers of health inequity (such as economic inequality and gaps in social protection, labor markets, taxation, structural discrimination, conflict, environmental degradation, and political power) remain marginal to most health strategies.

This gap between evidence and action raises a critical question: why has the proliferation of SDH research not led to more decisive and effective efforts to reduce health inequities? [9] Adverse political environments, austerity and declining commitment to solidarity are undeniably salient, especially the rise of political movements in powerful countries that question any focus on, or even redefine, equity [10]. However, focusing exclusively on politics risks overlooking how the nature, orientation, and practice of SDH research itself may have limited its policy relevance and transformative potential.

Here, we argue that deficiencies in prevailing approaches to SDH research have reduced its impact on health equity – that there are issues with what has been studied, how these studies are conducted, and who leads the research. While not discrediting the substantial and valuable body of existing work, we contend that dominant research paradigms, thematic emphases, and institutional practices constrain the ability of research to inform and support action. Drawing on literature reviews and deliberations undertaken to inform the 2025 WHO World Report on Social Determinants of Health Equity (with literature mostly drawn from the English language though discussions were held with a diverse group of participants across regions), we identify key gaps in the content, methods, and practice of SDH research, address counterarguments that locate the problem solely in politics, and propose directions for reorienting SDH research toward greater agency and impact.

## Thematic gaps

A central limitation of the SDH literature is its focus on problem identification and description [11]. Social epidemiology has been highly effective in documenting gradients in health outcomes by income, education, occupation, race, gender, and place, among other stratifiers [12]. These studies have been crucial in establishing that health inequities are systematic, socially determined, and avoidable. However, the dominance of descriptive research has also produced diminishing returns. In many contexts, the existence and direction of health inequities are no longer contested; what remains uncertain is how to reduce them.

There is a need to build a bridge between population-level evidence and individual clinical outcomes. While SDH research has robustly documented systemic inequities, the translation to individual-level implications remains underexplored, limiting its utility for clinicians and health systems practice. For example, population data showing how economic precarity amplifies chronic disease burdens must explicitly link to clinical metrics like glycemic control in diabetes or cardiovascular event rates in atrial fibrillation, where deprived individuals face 20–50% higher risks of adverse outcomes. This linkage would empower healthcare providers to integrate SDH screening into routine practice, informing personalized interventions (for example, tailored social prescribing) and policy advocacy, thereby transforming descriptive epidemiology into precision public health that narrows inequities [13, 14].

There is comparatively little research that rigorously examines policy measures capable of addressing social determinants at scale [15]. Questions such as which combinations of policies are most effective, under what conditions they work, and how they interact across sectors remain underexplored. Even less attention has been paid to strategies to overcome obstacles to implementation, including asymmetries of power in the political economy, fiscal constraints, institutional inertia and political resistance to change [16] Policymakers and communities are often left with detailed, compelling diagnoses of their equity challenges, but limited guidance on feasible courses of action.

Health inequities are produced and sustained by economic systems, power relations, and policy choices that advantage some groups over others [17]. Yet much SDH research treats these forces as background conditions rather than objects of analysis. There is insufficient examination of how to engage with and manage interests, ideologies, governance arrangements, and national and global economic structures. These forces shape both the distribution of social determinants and the policy space available to address them. While there is robust research diagnosing the contribution of these factors to health inequities, there is a lack of knowledge or engagement on how to act on them [18, 19]. Without this analysis, recommendations risk being technocratic and detached from real-world constraints.

There are a set of emerging determinants of health equity that require greater scholarly attention. Climate change, [20, 21] digitalization, [22, 23] commercial determinants of health, [24, 25] and increasing armed conflict [26]. are reshaping social and economic conditions in profound ways – with major health impacts. These dynamics intersect with each other and with existing inequities, often amplifying vulnerabilities [27]. However, research on these topics remains fragmented and insufficiently integrated into mainstream SDH frameworks, limiting the ability of health equity research to remain relevant in a changing global context. There is also the need for greater research in understanding multiple social determinants and their interactions, considering intersectionality, the impact of cumulative social determinants, and the multiple levels of social determinants (e.g., individual, household, community, societal).

Despite frequent rhetorical acknowledgment of “structural determinants,” there is insufficient research engaging with these structures in a meaningful and practical way, particularly to inform and support policy action [28]. Structural determinants are often operationalized through proximate indicators (such as income or education) without interrogating the structural forces (such as racism or sexism) that generate these gradients [29]. This approach risks depoliticizing inequity and obscuring the levers through which change might be achieved.

## Underused types of research and methods

The dominance of social epidemiology in SDH research also has methodological consequences. While epidemiological methods are well suited to identifying associations and gradients, they are less equipped to answer questions about causality in complex systems, policy design, and implementation pathways [12, 30]. As a result, SDH research has generated limited quantitative evidence on the effectiveness of solutions [31].

Policy and implementation research methods - such as health policy and systems research (including policy analysis), realist evaluation, and implementation science - remain underused in the SDH field [15, 32]. These approaches are designed to understand how interventions work in practice, how context shapes outcomes, and how policies can be adapted and scaled, including overcoming barriers to implementation. Their limited application has constrained learning about what it takes to translate SDH principles into sustained action. For example, exhaustive evidence linking income inequality to health inequities have not led to sustained redistributive policy in most high-income countries. In contrast, focused policy and implementation research on conditional cash transfers in several middle-income countries has contributed to policy implementation.

Modelling approaches also offer untapped potential [33, 34]. Simulation models, microsimulation, and systems dynamics modelling can be used to estimate the distributional impacts of policy options, explore trade-offs, and assess long-term equity implications. Yet such tools are rarely employed to consider SDH and policy options to act upon them.

These omissions are partly attributable to data gaps that undermine the evidence base. Almost twenty years after the CSDH’s third overarching recommendation to measure health inequities and monitor action on SDH, many countries lack routine, disaggregated data on health outcomes and social determinants by key equity stratifiers [4, 35]. This situation may now even deteriorate for many countries with the termination of the Demographic and Health Surveys (DHS) [36]. Where data exist, they are often fragmented across sectors and not linked in ways that allow for comprehensive analysis. These gaps limit both accountability and the ability to evaluate progress.

SDH research can champion community-based demonstration sites as quasi-experimental alternatives to randomized controlled trials (RCTs). RCTs are the gold standard for causality but are often impractical for structural SDH interventions due to ethical concerns, scale, and contextual variability—such as testing universal basic income or neighborhood revitalization across diverse settings. Demonstration sites, exemplified by Health Innovation Labs in South Africa, HIV community-led trials or Brazil’s Bolsa Família evaluations, offer rigorous, real-world evidence through longitudinal, mixed-methods designs [37, 38]. By prioritizing these scalable models, SDH research can generate transferable lessons, including on what works under resource constraints.

While there are risks that the use of artificial intelligence could worsen health inequities, its potential role, along with other advanced analytics including mathematical modelling, in addressing SDH research gaps has received limited attention [39]. While AI is not a panacea, it could support improved data integration, predictive modelling, scenario planning and policy simulation if applied thoughtfully and ethically. The absence of systematic exploration of these tools represents a missed opportunity.

## Deficiencies in research practice

Beyond what is studied and how, there are deficiencies in whom conducts SDH research and how it is – or more often, is not - embedded in social and political processes. Health equity and social determinants are inherently multisectoral, yet research is overwhelmingly led by health researchers and institutions [40]. This dominance can marginalize perspectives from economics, political science, sociology, psychology, organizational theory, and law - disciplines essential for understanding and influencing structural determinants. It can also mean that the interests of the health sector predominate over the interests of the other sectors that need to lead on action on social determinants – a phenomenon that has been termed “health imperialism” [41]. There is a need for broader training of researchers to transcend their silos to work collaboratively in a multi-disciplinary fashion across sectors to address the complexity of SDH.

Engagement with communities affected by health inequities is also often limited, with such communities rarely leading on SDH research. Many studies analyze communities as objects of inquiry rather than partners in knowledge production. This approach risks overlooking lived experience, local knowledge, and community-defined priorities, and can weaken the legitimacy and relevance of research findings [9]. This omission also ignores how communities have led action to reduce health inequities [42, 43], including the success of the HIV movement in increasing access to antiretroviral medication, with affected communities driving the knowledge agenda and policy action [44]. This model of leadership from affected communities has not been replicated widely in SDH research to address health inequities.

Similarly, engagement with policymakers is often insufficient or occurs too late in the research process. Research agendas are often driven by academic incentives rather than policy needs, and findings are disseminated in formats and venues aligned with academic incentives but often disconnected from decision-making processes. Research is rarely produced aligned to policy and budget cycles, with researchers understandably struggling to engage with the narrow political space and short-term time horizons of many policymakers. The result is a persistent gap between evidence production and policy uptake.

The academic orientation of SDH research also shapes its language and discourse, often ignoring practice-based approaches like the Healthy Cities movement. Technical terminology and normative ambiguity can make findings inaccessible to non-academic audiences, even if it can also ensure technical legitimacy that allows persistence in policy agendas even as political currents change. Media engagement is limited, and when it occurs, it is often episodic rather than strategic. Like much academic research, the complexity and mode of SDH research have struggled to persuade broad sections of the public health community – and is now also found wanting given the communication preferences of audiences increasingly accustomed to accessing knowledge through social media platforms and short videos, rather than long discursive text.

Finally, power dynamics within research itself warrant greater scrutiny. Funding priorities, authorship practices, and institutional hierarchies can reproduce global and local inequities, privileging voices from high-income settings and elite institutions [45]. These dynamics can undermine the emancipatory aspirations of health equity research. There has been a growing call to decolonize global health, including global health research [46]. While there is growing SDH research conducted and led by researchers in low- and middle-income countries, the field remains dominated by English language, high-income country researchers, or by elites within countries regardless of country income status [47]. Journal policies are evolving but still present barriers to the inclusion of knowledge from those beyond the academy and beyond the English language and of knowledge directly relevant to the community leadership and policy actions required to advance health equity.

## Is the problem politics rather than research conduct?

A reasonable response to the above critiques of SDH research may be that the fundamental constraint on progress is political rather than scientific. From this perspective, it might be argued that the evidence base on health inequities is already extensive, coherent, and compelling. What is lacking is not better research, but political will in contexts increasingly shaped by austerity, market-oriented policy paradigms, democratic backsliding, a reactionary backlash to attention to equity, and the growing influence of commercial and elite interests. If governments are unwilling or unable to act on redistributive policies, researchers may well argue that a change in their practice is unlikely to make a material difference.

This critique captures an essential truth: health inequities are produced and sustained through political choices, and research alone cannot overcome entrenched power relations. However, framing the issue as a binary choice between politics and research risks obscuring how knowledge production itself is embedded in political processes. Kingdon famously identified the three elements for a policy window for change – whereby a defined problem, an available and feasible solution, and a conducive political context or moment combine to result in change [48]. Politics is not an external force acting upon otherwise neutral evidence; rather, evidence shapes political agendas, frames what is considered feasible, and equips actors with arguments, narratives, and tools for contestation.

SDH research has contributed to authoritatively defining the problem of health inequity, but even this achievement is skewed to high-income countries. There is a much further way to go for SDH researchers to provide evidence on feasible mechanisms for implementation in current policy and fiscal environments - rather than proposing merely normative proposals. While analytically rigorous, much existing SDH research has struggled in generate greater impact partly because it often stops short of engaging with questions of prioritization, feasibility, sequencing, and surmounting opposition. Descriptive documentation of inequities, however morally compelling, can be politically inert when it does not confront the interests and institutions that benefit from the status quo nor illuminate actionable pathways. In some cases, repeated demonstrations of widening inequities without accompanying analysis of pathways for action may even contribute to fatalism among policymakers and advocates.

Research on SDH may not be able to overcome the primacy of politics but instead could seek to engage with it more directly and strategically. SDH researchers need to better link evidence to policy cycles and involve policymakers themselves more – to also contribute to advancing politically feasible moments through cultivating relationships with politicians and communities and even being able to explain why evidence-informed prescriptions will further a political agenda. Research that examines policy design, implementation constraints, and political economy dynamics can help identify leverage points for change, moments of opportunity, and strategies for coalition-building across sectors [49]. While such research will undoubtedly encounter resistance, it is better positioned to support actors working within and against political constraints than research that remains relatively abstract.

We appreciate the substantial body of SDH research that has contributed to agenda-setting, advocacy, and normative change over the past two decades. We acknowledge that there are good examples of SDH research that do engage with politics and have informed action [43]. And we do not suggest that evidence will automatically always translate into action if research practices are reformed. The question is not whether SDH research has been important, but rather whether it has sufficiently enabled the action required to catalyze transformative change. The status quo is not an option, particularly in a world with growing challenges to equity and solidarity – and where research funding itself is being increasingly challenged and politicized.

## Conclusion: Reorienting social determinants of health research for impact

In such a time, knowledge and science can be a crucial bulwark to combat forces arraigned against solidarity but must engage explicitly with the political conditions under which health equity is pursued or resisted. Evidence is only one ingredient to reform on SDH, but it remains a necessary ingredient. SDH data and research must be reoriented toward agency - understood as the capacity to inform, enable, and sustain action under real-world political and institutional constraints. SDH researchers must aim to create and cultivate policy windows of opportunity by being active protagonists in all three of Kingdon’s elements – to support or, in rare instances, even be the policy entrepreneurs that Kingdon highlighted as crucial for change [50]. Advancing agency for action on SDH requires changes not only in research topics, but also in how research is designed, conducted, and communicated.

First, SDH research must place greater emphasis on solutions – and even consider prioritization of these solutions. This involves systematic analysis of policy options across sectors, attention to implementation pathways, and explicit consideration of how interventions interact with existing institutions and power relations. Research should move beyond broad calls for action to specify which policies work, for whom, under what conditions, and at what cost. Embedding theories of change within research designs can help clarify how evidence is expected to contribute to equity outcomes. SDH research must also fully engage with areas of increasing importance, such as climate change and artificial intelligence, and consider the links between different determinants.

Second, greater diversity is needed in who produces and uses SDH research. Multisectoral challenges require multisectoral knowledge production. This includes stronger engagement across thematic sectors, with the health sector ceding leadership where appropriate, and meaningful collaboration with policymakers, practitioners, and civil society. Participatory and co-produced research approaches can enhance relevance, legitimacy, and accountability, particularly when they center the perspectives and leadership of communities experiencing health inequities.

Third, methodological pluralism should be actively promoted. Alongside social epidemiology, there is a need for expanded use of health policy and systems research, implementation science, political economy analysis, and modelling approaches capable of assessing distributional impacts and long-term scenarios. Addressing persistent data gaps - particularly through disaggregated and linked data systems - is essential. Emerging tools such as artificial intelligence may support these efforts if applied ethically and transparently, particularly ensuring addressing bias in large language models through considering the source texts through which models are trained. There is a need for broader training of researchers to transcend their silos to work collaboratively in a multi-disciplinary fashion across sectors to address the complexity of SDH.

Fourth, the communication of SDH research must be transformed. Evidence intended to influence policy and public debate should be conveyed in accessible, policy-relevant formats, supported by clear narratives about feasibility, trade-offs, and co-benefits. Strategic engagement with media, including social media, and decision-making processes should be treated as integral to research practice rather than as an afterthought. Scientific journals retain an important role, but there is also a need to publish in and benefit from journals in different sectors, such as environmental, political science, and urban planning journals. Notwithstanding risks, artificial intelligence has tremendous potential to facilitate customized communication products for different audiences from research programmes, though no communication can substitute for the essential engagement of policy actors described above.

Fifth and finally, the incentives for SDH research and researchers in academic institutions need to be reformed to facilitate their role in agency for SDH action, correcting current misalignments. Key to doing so is strengthening brokering institutions and individuals in countries who can link research and policy and prioritize intentional action towards policy impact in research rather than a sole focus on publications and reports as ends in themselves. Sustainable funding is also required for policy and interdisciplinary social determinants of health equity research to address current gaps and advance priorities going forward.

Reorienting SDH research in these ways will not, on its own, resolve the political challenges that underpin health inequities. However, in a period marked by overlapping crises and growing threats to equity and solidarity, research that remains focused on problem description alone is no longer sufficient. SDH research alone is not responsible for disappointing progress on health inequities, but it has constrained the range of potential action. A shift from evidence accumulation to agency-oriented research offers the best chance for knowledge to function as a meaningful resource in the ongoing struggle for health equity – and for researchers to contribute to policy windows that advance this struggle.

## Data Availability

No datasets were generated or analysed during the current study.
